# A meta-analysis of the association between mindfulness and motivation

**DOI:** 10.3389/fpubh.2023.1159902

**Published:** 2023-08-08

**Authors:** Li-ying Li, Xue Meng, Wen-ting Hu, Jia-sen Geng, Tian-hua Cheng, Jia-cheng Luo, Ming-yu Hu, Hai-yue Li, Yi Wang, Yan-yu Wang

**Affiliations:** ^1^School of Psychology, Weifang Medical University, Weifang, China; ^2^Neuropsychology and Applied Cognitive Neuroscience Laboratory, CAS Key Laboratory of Mental Health, Institute of Psychology, Beijing, China; ^3^Department of Psychology, University of Chinese Academy of Sciences, Beijing, China

**Keywords:** mindfulness, motivation, intrinsic motivation, extrinsic motivation, amotivation

## Abstract

**Introduction:**

Mindfulness reflects attention to the present moment in a non-judgmental way and has been linked to individual autonomy and motivation, but conclusions are inconsistent. The purpose of this review was to summarize previous studies to explore the relationship between mindfulness and motivation and its intervention effects.

**Methods:**

Literature searches were conducted in five electronic databases. Both correlational studies assessing the association between motivation and mindfulness and experimental studies to verify the effect of intervention were included.

**Results:**

Six papers with seven intervention studies and twenty-three papers with twenty-seven correlational studies met the inclusion criteria. Meta-analysis showed that mindfulness was positively correlated with intrinsic motivation (*r* = 0.28, *p* < 0.0001) and total motivation (*r* = 0.37, *p* < 0.0001) but had no significant correlation with extrinsic motivation (*r* = 0.01, *p* = 0.93) or amotivation (*r* = −0.17, *p* = 0.14). Effect-size estimates suggested that mindfulness intervention was beneficial to motivation promotion, but the effect was at a low level (*g* = 0.12).

**Conclusion:**

We found consistent support for mindfulness practice relating to motivation promotion, especially on intrinsic motivation development. However, there was still a portion of heterogeneity that could not be explained and needed to be identified in future studies.

## 1. Introduction

Motivation, defined as the psychological tendency or internal drive that stimulates and maintains the action of an organism, lies at the heart of all behavior of human being and the focus topic of psychology and pedagogy (1). An individual with a high level of motivation is more productive during their study or work. For example, academic achievement has been reported to be influenced by different types of motivation that stem from external incentives, ego involvement, personal value, and intrinsic interest (2). Motivation is also associated with individual participation in physical activity or maintaining a healthy lifestyle (3–5). Lack of motivation, on the other hand, is typically categorized as the syndrome of anhedonia (6, 7), which is often identified a central feature of some mental disorders (8), such as schizophrenia or major depressive disorder, and is strongly linked to individual poor functional outcomes (9). Finding from Barch et al. (10) showed that patients with strong anhedonia often exhibited motivation deficits. Therefore, how to improve individual motivation in order to maintain high work and study results or maintain a healthy lifestyle is of great significance for personal development.

Traditional psychologists defined motivation as the psychological disposition or drive that inspires and sustains an individual to perform an activity and leads to that activity toward a goal (11). As Ryan and Deci found in the 1970s that imposed extrinsic rewards had a debilitating effect on individuals’ interest, attention has been paid to the distinction between intrinsic and extrinsic motivation (12). Subsequently, many researchers proposed different theories about the relationship between intrinsic and extrinsic motivation. For example, Deci et al. (13) obtained consistent results from laboratory experiments with different subjects using different procedures and stimuli: extrinsic stimuli weakened pre-existing intrinsic motivation (14). Simon argued that the most important function of motivation is the control of attention, and the difference between intrinsic and extrinsic motivation can be seen as the difference between distraction and concentration of attention (14).

Although there is no conclusive theory on the relationship between intrinsic and extrinsic motivation, most researchers agreed that extrinsic motivation drives individuals when they engage in activities for the pleasure of obtaining external objects (e.g., money), whereas intrinsic motivation drives individuals when they engage in activities for the pleasure of the activity itself and to satisfy basic human psychological needs (e.g., autonomy) (12). Accordingly, why do people experience amotivation, perhaps because they do not see the connection between their behavior and the expected result, and/or feel incapable of doing the work. Amotivation is thus associated with theories concerning low expectancy and/or value (15), low self-efficacy (16), and learned helplessness (17).

Mindfulness reflects attention to the present moment in a non-judgmental, non-reactive manner (18, 19). Conceptually related to mindfulness is the construct of savoring (20), which means the attention to, appreciation, and enhancement of positive experiences in the moment (21). Some scholars further divide mindfulness into trait and state, with the former thought to be a personality trait and the latter thought to be cultivable (18, 22, 23). Through mindfulness practice, people learn to observe sensations and be more motivated in their daily life. Ryan and Deci (24) proposed that “mindfulness, defined as the open and receptive awareness of what is occurring both within people and within their context, facilitates greater autonomy and integrated self-regulation” (p. 268). Through attention-directed training, the trainers’ thought patterns can be changed which in turn leads to changes in attitudes and behaviors (25).

In everyday life, there is evidence of enhancements in both psychological and physical aspects of well-being and emotion regulation, following mindfulness training (26). Some studies have found that mindfulness-based intervention can significantly improve depressive mood, anxiety (27–29), sleep disturbance (30), cognitive function in older adults (31), even hedonic capacity among patients with chronic pain (32). However, some studies have found no such effect (33–35). Liu et al. (36) conducted one systematic review and found that mindfulness intervention had significant improvement effect on negative symptom, such as amotivation and anhedonia, among patients with schizophrenia. However, the question of whether mindfulness practices can increase levels of motivation and decrease amotivation in the broader population is unclear.

In addition, although some studies have found that mindfulness was significantly related to both extrinsic and intrinsic (5), the results of some experimental studies have only found that mindfulness improved intrinsic motivation (35–38). There are even studies that have found the opposite effect. For example, Marion-Jetten et al. (39) have found that higher levels of dispositional mindfulness had lower controlled goal motivation. Therefore, it is not clear whether mindfulness practice has a positive effect on both intrinsic and extrinsic motivation. Which motivation, if any, works better.

Taken together, although some previous studies have found that mindfulness practice has an effect on the improvement of motivation, some studies have not found this effect. In addition, there is no consensus on whether extrinsic motivation or intrinsic motivation is more closely related to mindfulness practice, and whether both can be improved by mindfulness practice.

Therefore, in this study, we conducted a meta-analysis combining existing correlational and intervention studies in an attempt to explore the relationship between mindfulness and motivation. Based on the existing studies (35–38, 40), we hypothesized that mindfulness would be significantly associated with motivation. We also hypothesized that mindfulness could improve amotivation and increase the level of motivation, especially in the case of intrinsic motivation.

## 2. Methods

### 2.1. Eligibility criteria

This systematic review was conducted and reported in accordance with the Preferred Reporting Items for Systematic Reviews and Meta-Analysis (PRISMA) guidelines (41). To be included, studies needed to meet the following criteria: (a) studies described as quantitative, not qualitative, measures of mindfulness and motivation; (b) studies examined mindfulness and motivation; (c) studies reported the relations between mindfulness and motivation, including either an effect size (e.g., Cohen’s *d*), or sufficient information to compute an effect size; (d) studies were included only if they were written in English and published as full-text articles in peer-reviewed professional journals; (e) studies used an intervention (with or without a control condition) or correlational design. The exclusion criteria were as follows: (a) mindfulness was not considered an element of intervention; (b) studies were not published in a peer-review journal in English; (c) data was unavailable to compute.

### 2.2. Search strategies

Literature searched were conducted in five databases, including PubMed, Embase, Cochrane Central Register of Controlled Trials, PsycINFO, Web of Science. The search was performed for articles published from the earliest available date to 30 September 2022.

We used the search terms “mindfulness” and “motivation”/“anhedonia.” The following search terms example were initially searched in PubMed: [Mindfulness*(Title/Abstract)] OR [MBSR(Title/Abstract)] OR [MBCT(Title/Abstract)] OR “[Mindfulness”(Mesh)] AND [“Anhedonia”(Mesh)] OR [“Motivation”(Mesh)] OR [anhedonia(Title/Abstract)] OR [amotivation(Title/Abstract)] OR [motivation(Title/Abstract)] OR [“negative symptom”(Title/Abstract)].

**Figure 1:**
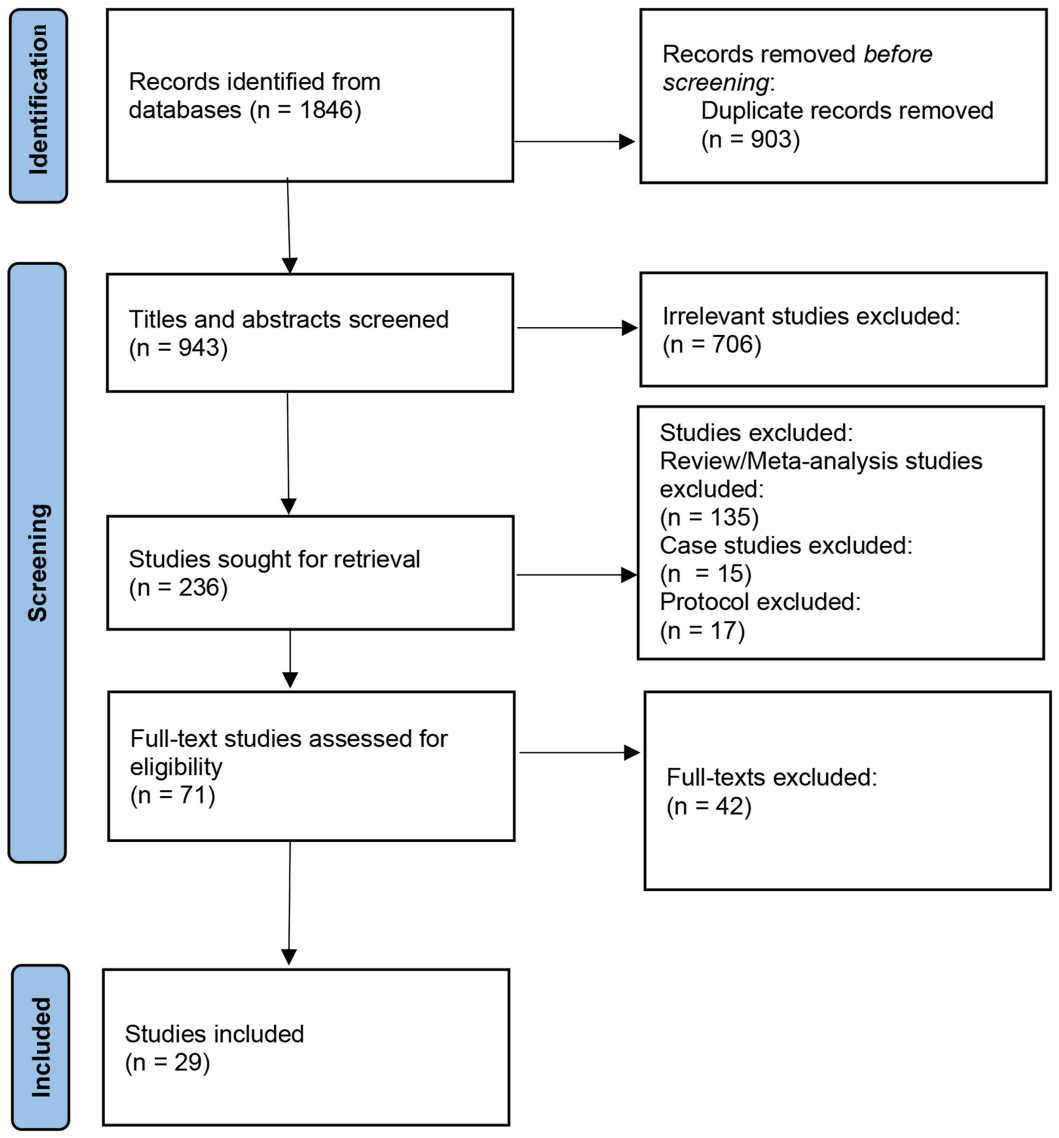
PRISMA flow-diagram showing the study selection process.

Titles and abstracts were screened. Four authors independently screened titles and abstracts of all the studies to exclude duplicate records, review papers, conference abstract and case studies. To confirm the inclusion, the same four authors assessed the eligibility of these full-texts and reasons for exclusion of publications of eligible studies were recorded. The details of the selection process were recorded to generate the PRISMA flow diagram (Figure 1).

### 2.3. Data extraction

Four researchers independently extracted data using self-designed data extraction forms from 29 papers included in this review. The forms included the following information: (a) title; (b) author(s); (c) publication year; (d) study design; (e) country of the participants; (f) participants’ demo-graphics; (g) number of participants (in each group, if intervention); (h) average age of participants; (i) proportion of female participants; (j) instrument used to measure mindfulness and motivation; (k) relevant outcomes; (l) intervention and control details (if intervention).

### 2.4. Synthesis

In this review, 27 correlation studies and 7 intervention studies were included, therefore outcomes were pooled separately. For correlational studies, all summary measures were converted to Fisher’s *z* and all analyses were performed in Fisher’s *z*. For intervention studies, all summary measures were converted to Hedges’ *g* (42), which corrects for biases due to small sample size from Cohen’s *d*. Appropriate data were pooled by using a random effects model and calculating the standardized mean difference. When studies report more than one time point, the latest time point which was chosen for analysis. All analyses were conducted in the R environment [Version 4.2.1 (43)] and meta-analyses were conducted using the *meta* package (44).

#### 2.4.1. Heterogeneity analysis

Heterogeneity was systematically assessed as the following steps: the generation of forest plots, the *I*^2^ statistic and chi-square [*Q* test (45)] and. *I*^2^ scores greater than 75% would be considered high heterogeneity, which could be explained by moderating factors, whereas 50% indicates moderate heterogeneity and lower than 25% indicates low heterogeneity (46).

#### 2.4.2. Assessment of publication biases

Publication bias was visually judged by the funnel plot (47). A symmetric distribution funnel illustrates no publication bias, while an asymmetrical funnel illustrates potential publication bias of the included studies. We further performed Egger’s test of the intercept (48) to further explore the publication bias of asymmetric funnel plots.

#### 2.4.3. Assessment of risk of bias

For correlational designs included in the review, we drew upon the methods outlined in the PRISMA statement. Risk of bias criteria were: (a) description of participants demographic; (b) evidence that the sample is representative of the population which it selected; (c) a valid measurement instrument of mindfulness; (d) a valid measurement instrument of motivation. Four researchers independently assigned 0 (absent described) and 1 (present described). A total score of 1 or less was considered high risk, 2–3 was considered moderate risk and 4 was considered low risk. For intervention designs, risk of bias was assessed by using JADAD scale. Four criteria from this scale were adapted and four researchers independently assigned 0 (absent described), 1 (inadequately described) or 2 (present described). A total score of 1–3 was considered high risk and 4–7 was considered low risk. Publication bias was also visually judged by the funnel plot (47). The overall level of quality evidence was assessed for each outcome by grading evidence according to the Grade of Recommendation, Assessment, Development and Evaluation Working Group (GRADE) (49).

#### 2.4.4. Sensitivity analysis

Sensitivity analysis (50) was performed to examine the extent to which studies judged to be at high risk of bias influenced the results. Both random effects and fixed effects models were rerun to assess how outcomes were affected. Pooled estimated effect sizes were calculated by removing one study individually to assess how each study affects the pooled estimates.

## 3. Results

### 3.1. Study selection

A total of 1,846 records were identified from five databases. After removing 903 duplicates, 943 records were screened out by titles and abstracts. 706 irrelevant studies, 135 reviews, 15 case studies and 17 protocols were excluded. The full-text publications were retrieved for the remaining 71 papers and were assessed for the eligibility criteria. This resulted in the identification of 29 papers for inclusion.

### 3.2. Study characteristics

Of the 29 papers included in the meta-analysis, 6 papers with 7 studies were intervention studies and 23 papers with 27 studies were correlational studies. Of the correlational studies, since different measurement instruments were selected by each study, thirteen studies measured intrinsic motivation, eleven studies measured extrinsic motivation, six studies measured amotivation and thirteen studies measured total motivation. Further information on each study, including sample size, sex and measurement instruments of mindfulness and motivation used, could be found in Table 1.

**Table 1:** Summary of included studies performing correlation analysis.

Author(s)	Sample size (*n*)	Mean age (y)	Male/female	Country	Measures instrument			
					Motivation	Mindfulness	Risk of bias scores	Risk of bias rating
Ali et al. (51)	428			China	15-item scale (52)	5-item scale (53)	4	Low
Ying et al. (54)	363		192/171	China	3-item intrinsic motivation (55); 3-item extrinsic motivation (56)	6-item scale (57)	0	High
Amemiya and Sakairi (3)	111	19.65	88/23	Japan	JSMS	AMQ	2	Moderate
Pan and Liu (58)	577		235/342	China	10-item questionnaire (59)	MTS-C	3	Moderate
Böge et al. (60)	79	42.29	54/24	Germany	PANSS	SMQ	4	Low
Chen et al. (61)	606	20.54	144/462	China	Self-improvement motivation scale (62)	SCS	4	Low
Ghanizadeh et al. (63)	221	26.98	47/174	Iran	19-item scale (64)	LMS	3	Moderate
Hutmacher et al. (4)	1,877	14.74	955/922	Luxembourg and Germany	PLOC-R; BREQ-II	FMI	4	Low
Dust et al. (65)	151	29.46	66/85	China	State-level motivational control scale (66)	5-item scale (67)	2	Moderate
Levesque and Brown (68)	78		19/59	Rochester	SDS; PLOC	MAAS	2	Moderate
Neace et al. (34)	188	19.83	30/158	Amazon	EMI-2	MAAS	3	Moderate
Tekin et al. (35)	182		179/3	Turkey	TMQ	MAAS	4	Low
Torok and Keri (69)	300	38	152/148	Hungary	sO-LIFE	MAAS	4	Low
Wu et al. (70)	101	20.7	72/29	Taiwan of China	APSI	CMAAS	3	Moderate
Ruffault et al. (38)	244	21	102/142	France	BREQ-II (French version)	MAAS	4	Low
Mihelič and Culiberg (81)	319	19.73	137/182	Slovenia	AMS	MAAS	3	Moderate
Montani et al. (33)	138	32.97	68/70	Canada	MWMS (French version)	MAAS (French version)	4	Low
	157	33.6	77/80	Canada	MWMS (French version)	MAAS (French version)	4	Low
Yusainy et al. (5)	411	20.2	120/290	Indonesia	TSQ	MAAS	3	Moderate
Bernstein et al. (71)	76	30	41/35	Israel	MASQ	MAAS	3	Moderate
Elphinstone et al. (37)	247	32.02	53/194	Australia	BPNS (72)	MAAS	3	Moderate
	578	26.77	280/298	Australia	24-item scale (73)	FFMQ-SF	3	Moderate
Marion-Jetten et al. (39)	137	22	92/45	Canada	PLOC	FFMQ-SF	4	Low
	85	24.19	35/50	Canada	PLOC	FFMQ (German version)	3	Moderate
	357	40.71	177/180	Canada	PLOC	MAAS	4	Low
Tabak et al. (74)	60	46.73	32/28	United States	BIS/BAS	FFMQ	4	Low
Thomas and Garland (32)	115	48.3	43/72	United States	SHAPS	FFMQ	4	Low

JSMS, the Japanese version of the sport motivation scale; AMQ, the athlete mindfulness questionnaire; MTS-C, mindfulness in teaching scale; PANSS, the positive and negative syndrome scale; SMQ, the Southampton mindfulness questionnaire; SCS, self-compassion scale; LMS, Langer mindfulness scale; PLOC, perceived locus of causality; BREQ-II, behavioral regulation toward exercise questionnaire; FMI, Freiburg mindfulness inventory; SDS, self-determination scale; MAAS, mindful attention awareness scale; EMI-2, exercise motivation inventory; TMQ, treatment motivation questionnaire; sO-LIFE, the Oxford-Liverpool inventory of feelings and experiences, short version; APSI, athletic psychological skills inventory; AMS, academic motivation scale; MWMS, multidimensional work motivation scale; TSQ, treatment self-regulation questionnaire for adequate physical activity; MASQ, mood and anxiety symptom questionnaire; BPNS, basic psychological needs scales; FFMQ-SF, five facet mindfulness questionnaire-short form; BIS/BAS, the behavioral inhibition and activation scales; SHAPS, Snaith–Hamilton anhedonia and pleasure scale.

Of the intervention studies, the studies were published from 2015 to 2021. For the study design, seven studies were two-arm RCTs with inactive-controlled design, four studies were two arm RCTs with a parallel intervention group and one study was one-group pretest-posttest design. Further information on each study could be found in Table 2.

**Table 2:** Summary of included studies performing mindfulness intervention.

Author(s)	Mean age (y)	Country	Participation of intervention *N*	Descriptions of the intervention group	Length of intervention	Instrument used to measure Motivation	Randomization	Concealment of allocation	Double blinding	Withdrawals and dropouts	JADAD score	Risk of bias rating
Brown et al. (75)		United States	19	Male-voice mindfulness training	9 min, 40 s	IMI	1	1	2	1	5	Low
Cox et al. (76)	20.46	United States	315	16 weeks yoga courses	16 weeks	BREQ-2	0	0	0	1	1	High
Moir et al. (77)	20.9	Netherlands	111	3 h introductory session, three follow-up 30 min sessions, and the provision of a three-CD set that had been used as a mindfulness resource	8 weeks	MSLQ	2	2	0	1	5	Low
Oberleiter et al. (78)	31	Germany	43	A German video (“What do these emojis mean?”; from ProSieben Germany, broadcast in the program Galileo)	10 min	G-SIMS	2	2	2	1	7	Low
Smyth and Milyavskaya (79)	21.15	Canada	103	A 10 min abbreviated version of a guided meditation	10 min	A 3-item scale	1	1	2	1	5	Low
	19.93	Canada	60	A 10 min abbreviated version of a guided meditation	10 min	A 3-item scale	1	1	2	1	5	Low
Zanesco et al. (80)	52	United States	26	Vipassana meditation	1 month	DSSQ	0	0	0	1	1	High

IMI, the 5-item interest/enjoyment subscale of the intrinsic motivation inventory; BREQ-2, the intrinsic motivation and identified regulation subscales (four items each) from the behavioral regulation in exercise questionnaire-2; MSLQ, the motivated strategies for learning questionnaire; G-SIMS, the German version of the situational motivation scale; DSSQ, Dundee stress state questionnaire.

### 3.3. Risk of bias

The risk of bias summary was presented in Tables 1, 2. In sum, among correlational studies, one study was high risk (3.70%), thirteen studies were moderate risk (48.25%) and thirteen were low risk (48.25%). Among intervention studies, two of seven studies were high risk (28.57%) and five studies were low risk (71.43%). The overall level of quality evidence in intervention studies shows that three studies were high quality, three were moderate quality and one was low quality. All intervention outcome measures for each study is listed in Supplementary materials.

### 3.4. Publication bias

**Figure 2:**
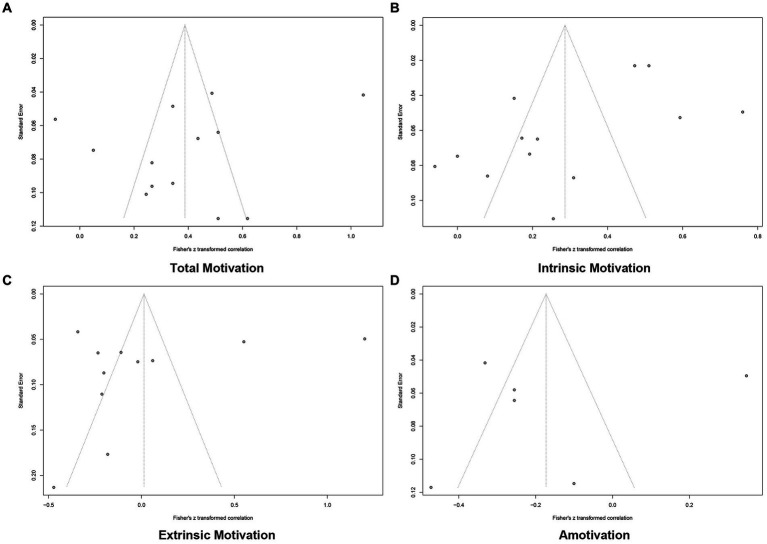
The funnel plots for correlational studies. (A) The funnel plot for total motivation in correlational studies; (B) The funnel plot for intrinsic motivation in correlational studies; (C) The funnel plot for extrinsic motivation in correlational studies; (D) The funnel plot for amotivation in correlational studies.

**Figure 3:**
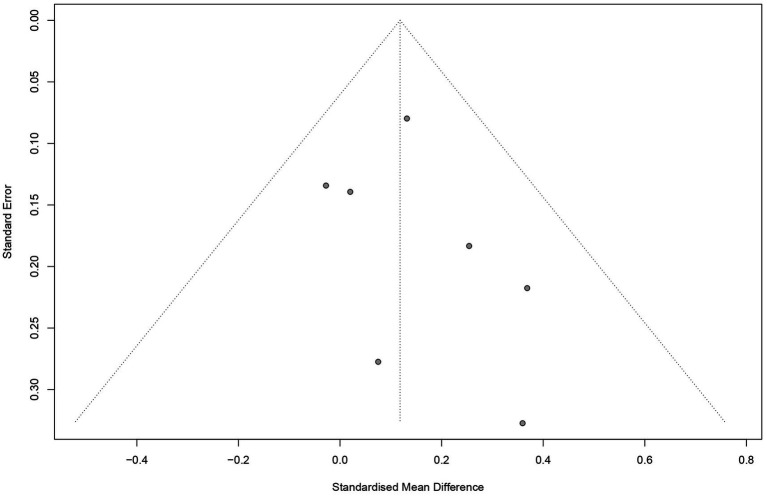
The funnel plots for intervention studies.

The funnel plots for correlational studies appear in Figure 2 and for intervention studies in Figure 3. Among the plots for correlational studies, the symmetry of the funnel plot was found to be good by examination of total motivation, however, there were clearly missing effect sizes for intrinsic motivation, extrinsic motivation and amotivation. Of the plots for intervention studies, there was a good symmetry, suggesting the likelihood of a risk of publication bias was low.

Based on the results of the visual inspections, we further performed an Egger linear regression test. For correlational studies, these tests indicated low levels of bias across all pooled effects: intrinsic (*t* = −2.43, *p* = 0.0335), extrinsic (*t* = −0.70, *p* = 0.5002), total motivation (*t* = −1.26, *p* = 0.2322). It was not possible to run Egger linear regression test for correlational amotivation studies and intervention studies because there were less than ten data points. Therefore, it can be concluded that there is no publication bias in this study.

### 3.5. Synthesis of results

#### 3.5.1. Correlational effects

**Figure 4:**
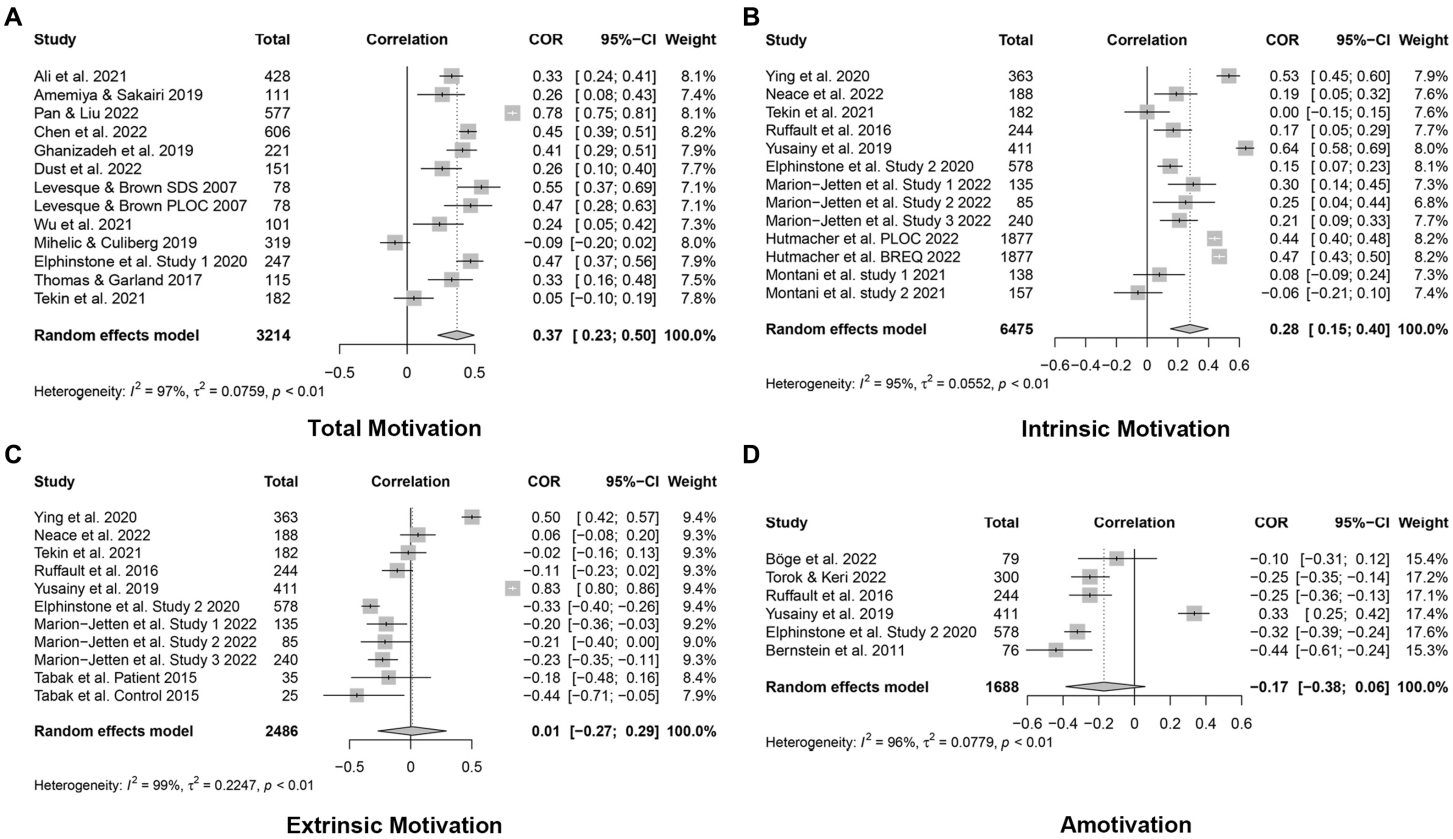
The forest diagrams for correlational studies. (A) The forest diagram for total motivation in correlational studies; (B) The forest diagram for intrinsic motivation in correlational studies; (C) The forest diagram for extrinsic motivation in correlational studies; (D) The forest diagram for amotivation in correlational studies.

In the results of total motivation, thirteen studies that measured the correlation between total motivation and mindfulness. These studies were found to be high heterogeneity (*Q* = 347.23, *p* < 0.0001, *I*^2^ = 96.5%). Therefore, the choice of random effects model is reasonable. The results showed that mindfulness had a moderate correlation with total motivation (*r* = 0.37, 95% CI = 0.23 to 0.50, *p* < 0.0001). The forest diagram is shown in Figure 4A.

In the results of intrinsic motivation, thirteen studies that measured the correlation between intrinsic motivation and mindfulness. These studies were found to be high heterogeneity (*Q* = 240.27, *p* < 0.0001, *I*^2^ = 95.0%). The results showed that mindfulness had a small correlation with intrinsic motivation (*r* = 0.28, 95% CI = 0.15 to 0.40, *p* < 0.0001). The forest diagram is shown in Figure 4B.

In the results of extrinsic motivation and amotivation, eleven studies measured the correlation between extrinsic motivation and mindfulness and six studies measured the correlation between amotivation and mindfulness. These studies were found to be high heterogeneity in extrinsic motivation (*Q* = 745.46, *p* < 0.0001, *I*^2^ = 98.7%) and amotivation (*Q* = 133.78, *p* < 0.0001, *I*^2^ = 96.3%). The results showed that a non-significant correlation between mindfulness with extrinsic motivation (*r* = 0.01, 95% CI = −0.27 to 0.29, *p* = 0.93) and amotivation (*r* = −0.17, 95% CI = −0.38 to 0.06, *p* = 0.14), respectively. The forest diagram is shown in Figures 4C,D.

The further subgroup analysis found that age and participant characteristics (i.e., clinical patients or not) could be considered as potential moderators of extrinsic motivation, the participants who were clinical patients (*g* = −0.16) and beyond 40 years (*g* = −0.24) evidenced lower effect size. A detailed description of subgroup analyses is in Section 3 of the Supplementary material.

#### 3.5.2. Intervention effects

**Figure 5:**
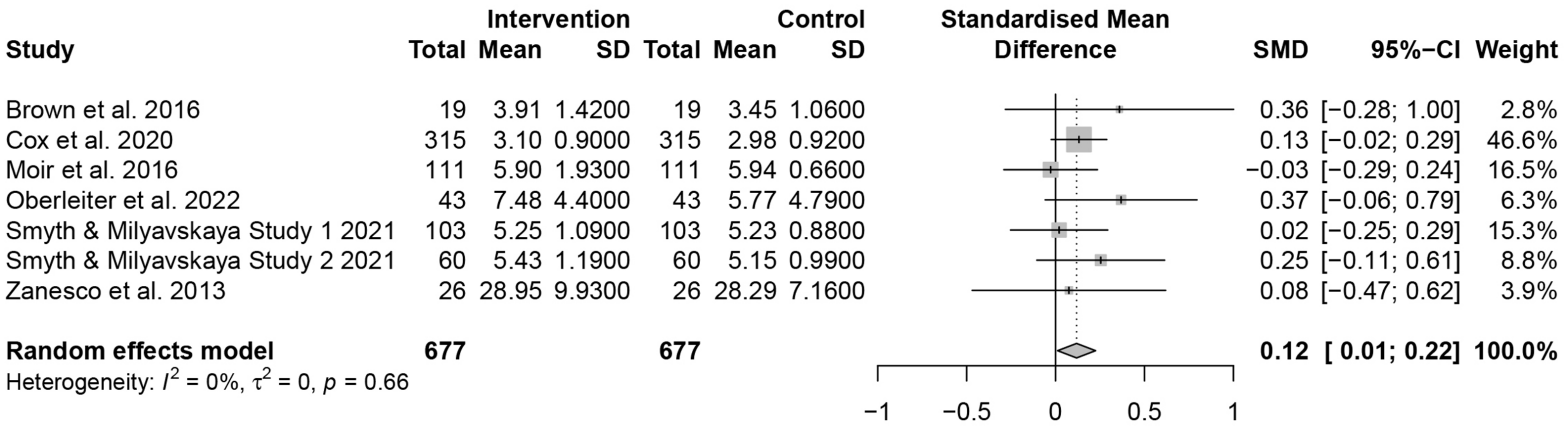
The forest diagram of motivation in intervention studies.

We included seven studies of mindfulness interventions in this review to test whether mindfulness promotes motivation. Figure 5 shows the pooled effects from studies of mindfulness interventions on motivation. These studies were found to be a low heterogeneity (*Q* = 4.14, *p* = 0.66, *I*^2^ = 0.0%). However, due to the small number of included studies, a random effect model still needs to be selected. We observed a low effect of mindfulness intervention on motivation promotion with a *g*-value of 0.12 (95% CI = 0.01 to 0.22, *p* < 0.05). A detailed description of full results is presented in Table 3.

**Table 3:** Estimated effect sizes between mindfulness and motivation.

Study design	Variable	*k*	*N*	Effect size (Hedges’ *g*/*r*)	Lower 95% CI	Upper 95% CI	*p*-value	*Q*	*I*^2^
Correlation studies	Motivation								
	Intrinsic motivation	13	6,475	0.2790	0.1525	0.3965	<0.0001	240.27	95.0%
	Extrinsic motivation	11	2,486	0.0135	−0.2665	0.2914	0.9265	745.46	98.7%
	Amotivation	6	1,688	−0.1712	−0.3840	0.0590	0.1441	133.78	96.3%
	Total	13	3,214	0.3693	0.2276	0.4957	<0.0001	347.23	96.5%
Intervention studies	Motivation	7	1,354	0.1182	0.0115	0.2239	0.0299	4.14	0.0%

*k*, the number of studies; *N*, the total number of participants.

### 3.6. Sensitivity analysis

Results from these models are reported in Supplementary materials and are summarized here. For correlational studies, no significant difference was found in the results of total motivation, intrinsic motivation and extrinsic motivation. In the results of amotivation, we found that Yusainy et al. (5) had an extreme value. If this study was omitted, the pooled correlation coefficient of mindfulness with amotivation would be −0.28 (95% CI = −0.34 to −0.22, *p* < 0.0001). In the other studies, no significant difference was found. The results of sensitivity analysis can be found in Supplementary Figures S1, S2.

## 4. Discussion

In the present review, we identified both correlational and intervention studies, a total of 34 studies, to investigate the relationship between mindfulness and different types of motivation and the effect of mindfulness training on motivational enhancement. After integrating the results of 27 correlational studies, we found that mindfulness was moderately correlated with total motivation. Across the dimensions of motivation, we found that mindfulness had a small positively correlation with intrinsic motivation, but no statistically significant with extrinsic motivation and amotivation. The analysis results of the experimental study further found the promotional effect of mindfulness training on motivation, although the effect size was at a small level. After visual inspections and Egger linear regression test, there was no publication bias in this review. However, due to the small sample size and the low level of evidence among intervention studies, the conclusions should be proposed with caution.

One of our main findings was mindfulness was moderately associated with motivation. This finding is consistent with some previous studies (3, 51), but there are still some studies that found no significant correlation between mindfulness and motivation (33, 81). This difference may be caused by several reasons. Firstly, there are many different theories about motivation. No uniform measurement instrument of motivation was accepted. Studies would be focus on different motivation scales, thus creating a gap between studies. Secondly, motivation can also be classified into different categories based on different theoretical backgrounds. Mindfulness may be effective in enhancing only one of these categories of motivation, but not all of them. Therefore, these studies may not have categorized different motivations when measuring motivation, leading to different results.

In our study, we explored the relationship between mindfulness on intrinsic and extrinsic motivation separately, and found that mindfulness was more closely associated with intrinsic motivation than extrinsic motivation. This supports our hypothesis and is consistent with some previous findings (39, 40). Compared with extrinsic motivation, intrinsic motivation is more emphasized individual autonomy, curiosity and interest (82). Autonomy/self-determination is the core element of intrinsic motivation (12). Extrinsic motivation, on the other hand, is caused by external rewards, such as money, material goods, and honors (12). Some studies argued that intrinsic motivation and extrinsic motivation are opposed to each other (14, 83). While only a weak negative correlation between intrinsic and extrinsic motivation was found in some studies (83). Mindfulness is considered as an intraindividual factor to support autonomous engagement in activities (24). The primary role of mindfulness was to enhance the individual’s attention and awareness to internal experience, including awareness of emotions, somatic states and psychological needs (84, 85). This is in line with the characteristics of intrinsic motivation. We believe that individuals with a high level of mindfulness are better able to become aware of their needs and thus improve their motivation level by increasing their autonomy. The closer association of mindfulness with intrinsic motivation further suggested the potential role of mindfulness practice on emphasizing the arousal of individual needs rather than the desire for external rewards.

Another main finding of our study was that mindfulness-based intervention could effectively improve individuals’ levels of motivation. Previous studies on the possible causes of mindfulness intervention on motivation can be summarized as follows. Firstly, the mindfulness-based intervention was found to be effective in improving the attention of individuals (86, 87). Smart et al. (86) found that after 8 weeks of mindfulness-based intervention, the subjects showed better self-awareness and improved immediate regulation of attention. This helps individuals to improve their self-determination, pay more attention to internal feelings, and reduce the interference of extraneous factors, thus promoting higher levels of motivation. Secondly, some researchers think that the effect of mindfulness-based intervention may be mediated by improving emotions to increase motivation levels. For example, unpleasant emotional experiences, such as anxiety, depression and nervousness, has been confirmed to seriously affect an individual’s social functioning and cause loss of interest in daily life (88). Some studies showed that mindfulness-based intervention was more effective in controlling anxiety symptoms in people with anxiety disorders (89–91). Mindfulness-based intervention can also reduce symptoms associated with major depressive disorder and increase an individual’s interest in life, thereby promoting increased motivation (92). Finally, some brain imaging studies have also found possible effects of mindfulness interventions on brain function. For example, Zhou and Liu (93) found that mindfulness-based intervention can enhance individual left-sided brain activity. In the study of Wang and Huang (94), mindfulness-based intervention was found to be related with the thickened gray matter of some brain areas, such as the hippocampus, insula and cingulate gyrus. Therefore, the intervention effect of mindfulness practice on motivation enhancement may have its cognitive neural mechanism, but relevant studies are relatively few, let alone the lack of intervention studies on the long-term effect on brain function.

This study has several limitations. Firstly, due to the limitation of the number of articles, we failed to investigate the correlation between different categories of mindfulness and different categories of motivation. Secondly, among the correlational studies, there was unexplained heterogeneity in pooled effects of mindfulness on some types of motivation, which may affect the analysis results. Thirdly, some articles did not select existing and accepted scales for motivation measurement and the degree of consistency of the instruments was not reported in the articles, which may lead to inaccurate motivation measures. Fourthly, only studies publication in English was included. Then, in the intervention study, we found by sensitivity analysis that if the non-RCT study was removed, the overall effect size became non-significant, although the *g*-value did not change significantly. This is likely to be due to the small number of studies that could be included. Lastly, the risk of bis assessment found a high risk of bias for two of seven intervention studies. Both were nonrandomization of participants, non-concealment of allocation and nonblinding of participants and researchers. These potential methodological deficiencies may affect the conclusions of the intervention studies in this review.

Despite the above limitations, this study is the first to analyze the relationship between mindfulness and motivation and the effect of intervention by integrating correlation studies and intervention studies. Although our findings may indicate that trait mindfulness and systematic mindfulness intervention could improve the level of individual motivation, further more intervention designs still need to be studied in different type of motivation to verify this finding. In addition, Mindfulness was more closely associated with intrinsic motivation than extrinsic motivation. Our study further confirms that mindfulness practice, a low-cost, easy-to-implement and productive daily training, has the potential to increase motivation levels in individuals’ daily lives and thus improve the quality of life. Future studies should pay more attention to the long-term effect of mindfulness intervention on motivational enhancement and its intrinsic neural mechanism.

## Data availability statement

The original contributions presented in the study are included in the article/Supplementary material, further inquiries can be directed to the corresponding author.

## Author contributions

Y-yW and YW designed the study and deeply modified the manuscript. L-yL performed the statistical analysis and wrote the first draft of the manuscript. XM and W-tH analyzedthe data andmodified the manuscript.J-sG, T-hC, J-cL, M-yH, and H-yL read, selected, and evaluated previous articles. All authors contributed to the article and approved the submitted version.

## Funding

This study was supported by a grant from the Natural Science Foundation of Shandong Province (ZR2021MC103), the Humanities and Social Science Research Project, Ministry of Education, China (19YJA190006), the Postgraduate Tutor Guidance Ability Improvement Project of Shandong Province (SDYKC20147) and the Science and Technology Project of Weifang Medical University.

## Conflict of interest

The authors declare that the research was conducted in the absence of any commercial or financial relationships that could be construed as a potential conflict of interest.

## Publisher’s note

All claims expressed in this article are solely those of the authors and do not necessarily represent those of their affiliated organizations, or those of the publisher, the editors and the reviewers. Any product that may be evaluated in this article, or claim that may be made by its manufacturer, is not guaranteed or endorsed by the publisher.
