# Supplementary material for: A meta-analysis of the association between mindfulness and motivation
Source: Front Public Health. 2023 Aug 8;11:1159902. doi: 10.3389/fpubh.2023.1159902 (PMC10442577; doi:10.3389/fpubh.2023.1159902)
Supplement: Supplementary file 1 [file Data_Sheet_1.pdf]

Supplementary Material

Mindfulness and Motivational Promotion: A Systematic Review and Meta-analysis of Clinical Trials

1 Risk of bias

Table 1s. Summary of overall level of evidence quality in intervention studies

Author(s)	Risk of bias	Inconsistency	Indirectness	Imprecision	Publication bias	Quality of evidence
Brown et al.				✓		⊕⊕⊕◯
Cox et al.	✓		✓			⊕⊕◯◯
Moir et al.		✓				⊕⊕⊕◯
Oberleiter et al.						⊕⊕⊕⊕
Smyth & Milyavskaya						⊕⊕⊕⊕<br>⊕⊕⊕⊕
Zanesco et al.	✓					⊕⊕⊕◯

⊕⊕⊕⊕High quality: We are very confident that the true effect lies close to the estimated effect.
⊕⊕⊕◯Moderate quality: We are moderately confident in the effect estimate.
⊕⊕◯◯Low quality: Our confidence in the effect estimate is limited.
⊕◯◯◯Very low quality: We have very little confidence in the effect estimate.

2 Sensitivity analysis

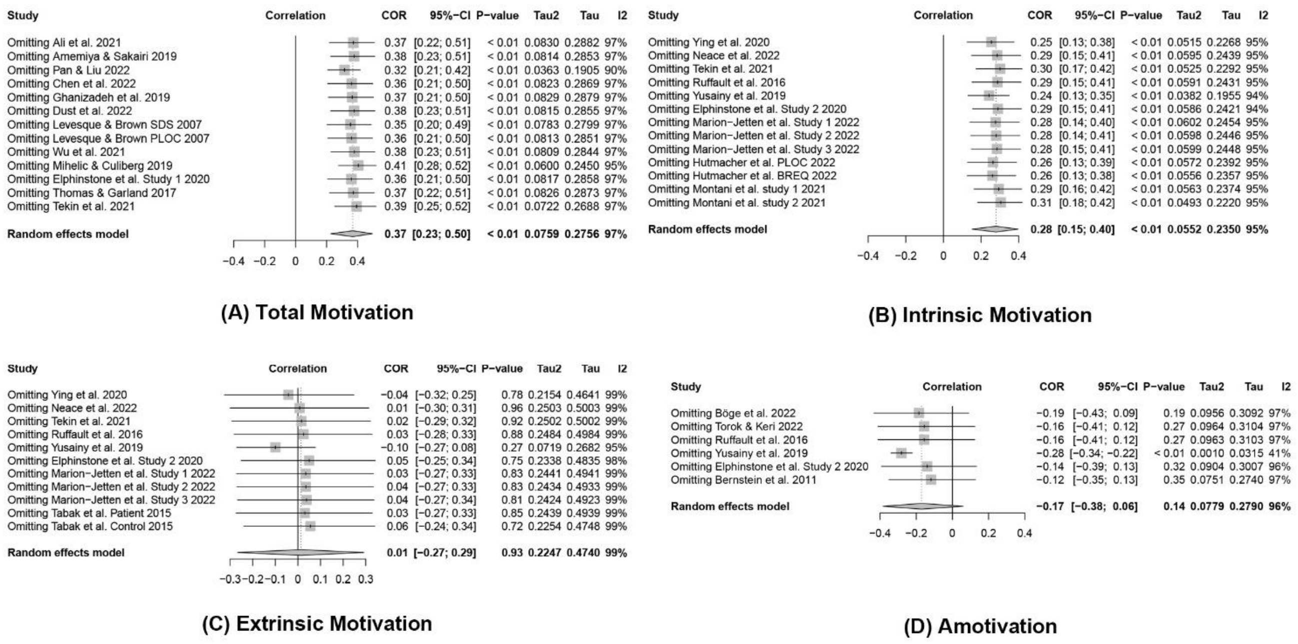

Figure 1s. The sensitivity analysis of correlational studies

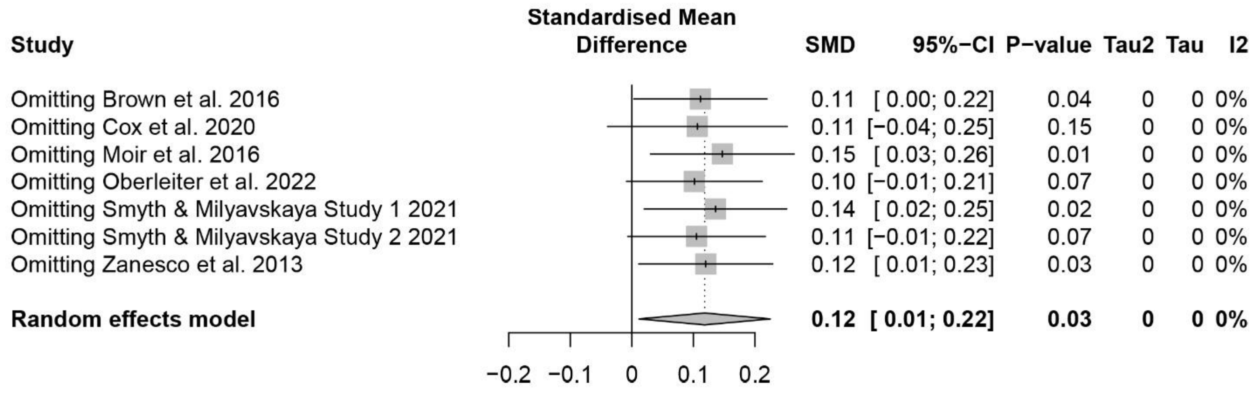

Figure 2s. The sensitivity analysis of intervention studies

3 Subgroup analysis

Table 2s. Subgroup analysis on intervention and correlational studies

		Participants	Effect size			Heterogeneity	
	k	N	*g/r*	95% lower	95% upper	Q	I^2
**Intervention studies**							
**Age**							
> 40 years	6	1058	0.12*	0.01	0.23	4.12	0.00%
< 40 years	1	50	0.08	-0.47	0.62	-	-
**Length of intervention**							
> 1 month	2	590	0.09	-0.05	0.23	1.04	4.00%
< 1 month	5	518	0.17	-0.01	0.34	2.65	0.00%
**Correlational studies**							
**Total motivation**							
**Age**							
> 40 years	2	346	0.6*	0.01	0.88	46.25	97.80%
< 40 years	11	2522	0.37***	0.22	0.51	345.68	96.80%
**Occupation**							
Athletes	2	212	0.25***	0.12	0.37	0.02	0.00%
Students	7	1977	0.37***	0.22	0.51	87.32	93.10%
Patients	2	297	0.19	-0.10	0.44	5.91	83.10%
Others	2	728	0.58	-0.10	0.89	71.45	98.60%
**Intrinsic motivation**							
**Occupation**							
Students	8	5395	0.34***	0.20	0.47	134.58	94.80%
Patients	1	182	0.00	-	-	-	-
Others	4	898	0.21	-0.07	0.45	59.17	94.90%
**Extrinsic motivation**							
**Age**							
> 40 years	3	300	-0.24***	-0.35	-0.13	1.29	0.00%
< 40 years	8	2186	0.26	-0.24	0.44	693.53	99.00%
**Occupation**							
Students	6	1641	0.06	-0.37	0.48	635.47	99.20%
Patients	3	242	-0.16	-0.39	0.08	4.36	54.10%
Others	2	603	0.16	-0.54	0.73	88.03	98.90%
**Amotivation**							
**Occupation**							
Students	3	1233	-0.08	-0.46	0.33	118.60	98.30%
Patients	2	155	-0.28	-0.57	0.08	5.15	80.60%
Others	1	300	-0.25	-	-	-	-

Note: k, number of studies; N, number of participants; *p < .05; **p < .01; ***p < .001.
